# The Functional Roles of MDSCs in Severe COVID-19 Pathogenesis

**DOI:** 10.3390/v16010027

**Published:** 2023-12-23

**Authors:** Jia Soon Len, Clara W. T. Koh, Kuan Rong Chan

**Affiliations:** 1 School of Biological Sciences, Nanyang Technological University, Singapore 637551, Singapore; jlen001@e.ntu.edu.sg; 2 Programme in Emerging Infectious Diseases, Duke-NUS Medical School, Singapore 169857, Singapore; clara.koh@duke-nus.edu.sg

**Keywords:** SARS-CoV-2, COVID-19, myeloid-derived suppressor cells, infection, innate immunity, T cells

## Abstract

Severe COVID-19 is a major cause of morbidity and mortality worldwide, especially among those with co-morbidities, the elderly, and the immunocompromised. However, the molecular determinants critical for severe COVID-19 progression remain to be fully elucidated. Meta-analyses of transcriptomic RNAseq and single-cell sequencing datasets comparing severe and mild COVID-19 patients have demonstrated that the early expansion of myeloid-derived suppressor cells (MDSCs) could be a key feature of severe COVID-19 progression. Besides serving as potential early prognostic biomarkers for severe COVID-19 progression, several studies have also indicated the functional roles of MDSCs in severe COVID-19 pathogenesis and possibly even long COVID. Given the potential links between MDSCs and severe COVID-19, we examine the existing literature summarizing the characteristics of MDSCs, provide evidence of MDSCs in facilitating severe COVID-19 pathogenesis, and discuss the potential therapeutic avenues that can be explored to reduce the risk and burden of severe COVID-19. We also provide a web app where users can visualize the temporal changes in specific genes or MDSC-related gene sets during severe COVID-19 progression and disease resolution, based on our previous study.

## 1. Introduction

The COVID-19 pandemic was caused by SARS-CoV-2 infections, which led to over 670 million cases and approximately 6.8 million deaths worldwide [1]. The virus genome encodes for 4 major structural proteins (nucleocapsid (N), spike (S), envelope (E), and membrane (M) proteins), 16 non-structural proteins, as well as several accessory proteins [2]. SARS-CoV-2 S protein comprises two subunits: S1, which is responsible for binding to the angiotensin-converting enzyme 2 (ACE2), and S2, which facilitates membrane fusion between the virus and the membrane of the target cell [3,4,5]. SARS-CoV-2 infections are often asymptomatic, although symptomatic infections can lead to a broad range of clinical outcomes, which can range from mild to severe outcomes [6,7]. The elderly [8], those with comorbidities [9], and the immunocompromised [10] are at the greatest risk of severe COVID-19, which is frequently characterized by hyperinflammation and cytokine storm [11,12], where markedly elevated levels of circulating proinflammatory cytokines can potentially lead to multi-organ damage and acute respiratory distress syndrome (ARDS). Other factors such as pre-existing immunity [13], gender [14], ethnicity [15], and diabetes [16] have also been identified as risk factors for severe COVID-19. Interestingly, based on our meta-analysis of RNAseq data across seven independent cohorts, time-series analysis, and single-cell RNA sequencing datasets, we previously demonstrated that the differential expression of *MCEMP1* and *HLA-DRA* in CD14-positive cells could be detected as early as 4 days before the nadir of respiratory function. As the upregulation of *MCEMP1* and downregulation of *HLA-DRA* are characteristic of myeloid-derived suppressor cells (MDSCs), the results indicated that an early expansion of MDSCs could be involved in severe COVID-19 progression and pathogenesis [17]. Besides our study, emerging work from other groups has also documented the contribution of MDSCs in severe COVID-19 pathogenesis [18,19]. Therefore, besides serving as an early prognostic biomarker for severe COVID-19, MDSCs could potentially be targeted to reduce disease burden. In light of these studies, we examine the current literature documenting the potential functional roles of MDSCs in severe COVID-19 and long-COVID pathogenesis, discuss strategies that can be employed to detect MDSCs, and explore potential therapeutic avenues to target MDSCs to reduce the risk of severe COVID-19.

## 2. MDSC Development and Functions

MDSCs was a term first introduced by Gabrilovich, D. I. et al. in 2007 [20] as an effort to describe a heterogeneous group of immature myeloid cells possessing potent immunosuppressive activities that are implicated in cancer pathology. MDSCs can be broadly subdivided into two main groups: granulocytic/polymorphonuclear MDSCs (PMN-MDSCs) and monocytic MDSCs (M-MDSCs), based on their lineage association [21,22]. The third subgroup, termed early MDSCs, makes up the minority of MDSCs and primarily comprises myeloid precursors and progenitors.

MDSCs arise from hematopoietic stem cells (HSC) via granulo-monocytic precursors (GMPs). GMPs differentiate from the common myeloid progenitor (CMP). A two-signal model [23] is widely accepted for the development of MDSCs. The first signal involves the expansion of immature myeloid cell populations by G-CSF, GM-CSF, M-CSF, and transcriptional regulators such as IRF8, STAT3, STAT5, NOTCH, and C/EBP-β [24]. This initial signal is thought to allow the expansion of immature myeloid cells while preventing terminal differentiation [21,22,24]. Thereafter, the second signal involves cues from the extracellular environment such as damage-associated molecular patterns (DAMPs), S100A8/A9, and proinflammatory cytokines (e.g., IL-1β, tumor necrosis factor, interferon-γ (IFN-γ), IL-4, IL-6, IL-13) in the peripheral tissues, which have been proposed to activate immature myeloid cells to gain immunosuppressive capabilities [22,24].

MDSC development occurs through the same differentiation pathways as neutrophils and monocytes [22]. Under normal physiological conditions, myelopoiesis is driven by GM-CSF while differentiation to granulocytes and macrophages is induced by G-CSF and M-CSF, respectively. Notably, while the differentiation to MDSCs, neutrophils, and monocytes requires GM-CSF, M-, and G-CSF, the requirement for these factors is higher for MDSCs compared to neutrophils and monocytes. Thus, MDSCs usually do not accumulate under normal physiological conditions [21,22,25]. However, during cancer development, chronic infections, and other pathological conditions, normal myelopoiesis may be perturbed [21,22,24], as these conditions can provide prolonged low-strength signals to persistently stimulate the myeloid compartment through the excessive production of signals such as GM-CSF, IL-6, IL-8, TNF-α, IL-1β, S100A8/9, and reactive oxygen species (ROS) [26] that are required for MDSC development. Emerging studies also support the fact that these signals are greatly induced after infections by viruses such as SARS-CoV-2 [27], HIV [28], and hepatitis B virus [29], which can aid MDSC development. However, the underlying mechanisms for MDSC generation may differ under different pathological conditions [30].

**Figure 1:**
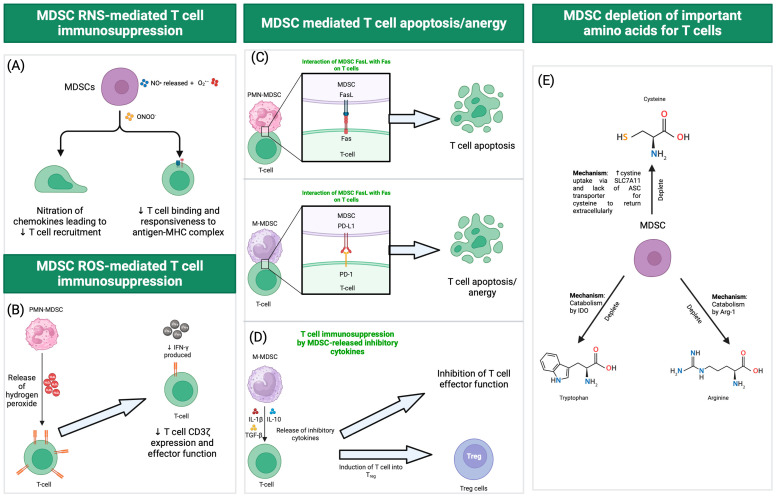
(**A**) MDSCs can produce reactive nitrogen species (RNS) such as nitric oxide through inducible nitric oxide synthase (iNOS). These NO^•^ released by MDSCs can react with superoxide (O_2_^•−^) to form peroxynitrite (ONOO^−^). Peroxynitrite can lead to the nitration of chemokines (e.g., CCL2) and proteins involved in the TCR signaling cascade (e.g., lymphocyte-specific protein tyrosine kinase) which hinders T cell recruitment and activation. Peroxynitrite can also reduce T cell binding and responsiveness to antigen–MHC complex through nitration of tyrosines (forming nitrotyrosine) in TCR-CD8 complex. (**B**) MDSCs can produce reactive oxygen species (ROS) such as superoxide (O_2_^•−^) through NADPH oxidase 2 (NOX2). Release of hydrogen peroxide (H_2_O_2_) from MDSCs has been shown to reduce T cell CD3ζ expression and inhibit T cell effector function such as the production of IFN-γ. (**C**) Interaction between Fas expressed on PMN-MDSCs with FasL on T cells induces apoptosis of cytotoxic T cells. M-MDSCs can also induce T cell apoptosis or anergy through PD-L1 which binds PD-1 on T cells. (**D**) M-MDSCs can release inhibitory cytokines such as IL-1β, IL-10, and TGF-β to inhibit T cell activation and function. (**E**) MDSCs can deplete amino acids, such as cystine/cysteine, which are essential for T cells. Uptake of cystine through SLC7A11 on MDSCs results in the conversion of cystine to cysteine within the cell. As MDSCs do not express the ASC transporter, cysteine cannot be released from the cell. MDSCs can also deplete the availability of tryptophan for T cells through IDO, which catabolizes tryptophan to N-formylkynurenine. Finally, MDSCs can deplete the availability of arginine for T cells through Arg-1, which catabolizes arginine to L-ornithine and urea. SLC7A11: solute carrier family 7 member 11. ASC: alanine-serine-cysteine transporter. IDO: indoleamine 2,3-dioxygenase. Arg-1: Arginase-1. Much of the immunosuppressive function of MDSCs has been uncovered from cancer studies and may not always be extrapolated to other contexts.

MDSCs are capable of suppressing various immune cell types such as B, NK, and, in particular, T cells [31]. MDSCs can exert their immunosuppressive activities [21,22,24,32] through the depletion of arginine, tryptophan, and cystine/cysteine availability, the induction of T_reg_ cells, RNS/ROS, the induction of T cell apoptosis via Fas/FasL interaction, the production of inhibitory cytokines, and PD-1/PD-L1 interaction [33] (Figure 1). Interestingly, many of these mechanisms involve immunometabolic regulation. For instance, arginine depletion by MDSC can be catalyzed by arginase-1 (Arg-1), which catabolizes arginine into L-ornithine and urea, reducing the availability of arginine and compromising T cell proliferation and function by reducing the expression of the CD3ζ chain [34]. In addition, MDSCs can deplete tryptophan availability for T cells by upregulating indoleamine 2,3-dioxygenase, catabolizing tryptophan to *N*-formylkynurenine, resulting in the inhibition of effector T cell proliferation while favoring the thriving of Foxp3^+^ T_reg_ cells. Cysteine, another amino acid important for T cell function, is also known to be sequestered by MDSCs which express SLC7A11, resulting in the uptake of cystine into the cell [35]. Because MDSCs do not express the alanine-serine-cysteine (ASC) transporter, the extracellular return of cysteine is prevented [35].

Besides immunometabolic regulation, MDSCs can generate ROS to mediate immunosuppression [26]. The elevated production of superoxides can react with nitric oxide to form peroxynitrite, which leads to the nitration of TCR-CD8, which has been shown to hamper the ability of CD8^+^ T cells to bind and respond to the peptide-major histocompatibility complex (pMHC) [36]. In addition, MDSC-like cells have been reported to release H_2_O_2_ to inhibit T cell activation and rely on superoxide generation to suppress T cell responses in a NOX2-dependent manner. ROS are also important mediators employed by MDSC for the immunosuppression of NK and B cells [26]. Within MDSCs, some of these immunosuppressive strategies are preferentially used by the PMN-MDSC subset (arginase-1, ROS, peroxynitrite) while others are used by the M-MDSCs subset (nitric oxide, PD-L1, and immunosuppressive cytokines) [21].

## 3. MDSC Identification and Characterization

While definitive strategies for identifying MDSCs are lacking, several combinations of cell markers have been suggested, namely CD11b^+^CD14^−^CD15^+^ or CD11b^+^CD14^−^CD66b^+^, for characterizing PMN-MDSCs and CD11b^+^CD14^+^HLA-DR^−/lo^CD15^−^ for the characterization of M-MDSCs in human peripheral blood mononuclear cells [37]. M-MDSCs can be distinguished from monocytes by low or undetectable HLA-DR protein expression. However, discriminating between PMN-MDSCs and neutrophils is more challenging, as their phenotypic markers and functions overlap. For instance, activated neutrophils can also secrete arginase-1 (Arg-1) and employ reactive oxygen species (ROS) to suppress T cell activity, akin to the function of PMN-MDSCs [38,39]. While more research will be required to better distinguish PMN-MDSCs from neutrophils, a possible approach could be to separate them by density, as PMN-MDSCs appear to be of a lower density compared to neutrophils [21,37].

Nonetheless, with the advent of single-cell RNA sequencing, PMN-MDSCs and M-MDSCs can be potentially characterized by transcriptomic signatures (Table 1). Although phenotypic cell markers specific to MDSCs are lacking, MDSCs have been found to have transcriptional profiles distinct from classical neutrophils or monocytes. M-MDSCs express proinflammatory genes and genes involved in mediating immunosuppression, such as NOS2, IL10, TNF, WFDC17, STAT3, S100A8/9, IL6, ARG1/2, CD84, TGFB1, and VEGFA. On the other hand, gene signatures for PMN-MDSCs include IL1B, IL4R, STAT1, STAT3, STAT6, OLR1, ARG1/2, PTGS2, S100A8/9, IRF1, LYZ2, TREM1, CXCL1, LILRA3, CSF1, IL6, and ANXA1, which allows them to be distinguished from classical neutrophils.

In both M- and PMN-MDSCs, STAT3 is upregulated and is important for expansion while preventing further differentiation to other cell types. For instance, it has been demonstrated that the inhibition of STAT3 expression abrogated MDSC expansion induced by tumor-derived factors [40]. Furthermore, the binding of MDSC-promoting signals (e.g., IL-6, G-CSF, GM-CSF, and M-CSF) to their respective cellular receptor activates JAK-STAT3 signaling, leading to the accumulation and expansion of MDSCs [41]. Activated (phosphorylated) STAT3 form homo- or heterodimers that can subsequently translocate to the nucleus and modulate gene expression by downregulating IRF8 expression and promoting the expression of S100A8/9, which is required for MDSC expansion and preventing the differentiation of MDSCs (reviewed by [42]). In addition to IRF8 downregulation, MDSCs also exhibit an increased expression of C/EBP-β (a member of the family of basic region leucine zipper transcriptional factor) which favors MDSC generation. IRF8 is a negative regulator of MDSC expansion while C/EBP-β, a master regulator of emergency myelopoiesis, also regulates arginase-1 and inducible nitric oxide synthase (iNOS) important for MDSCs to exert immunosuppressive effects [22]. Other gene signatures, such as WFDC17, ARG2, IL1B, and CD84, have also been found to be expressed by both M- and PMN-MDSCs [21,24].

**Figure 2:**
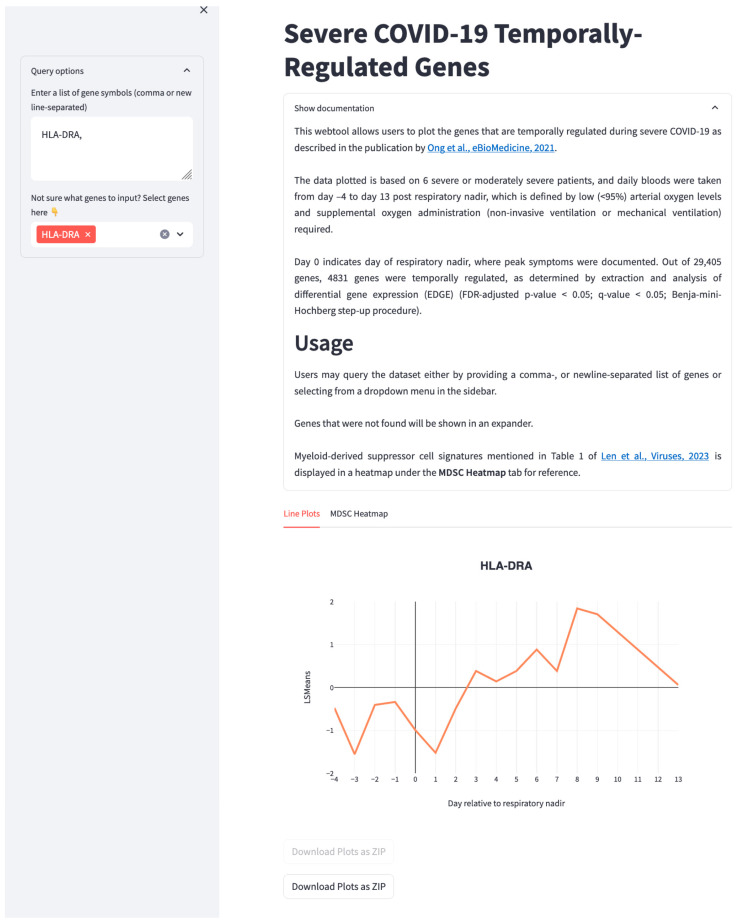
Web tool demonstrating how users can query individual genes or MDSC-related gene sets (the query gene shown here is HLA-DRA) to examine their temporal dynamics across multiple time points before and after the respiratory nadir. Respiratory nadir refers to the patient condition whereby arterial oxygen levels are low (<95%) and patients require supplemental oxygen administration (non-invasive ventilation or mechanical ventilation). Data based on Ong et al., 2021 [43]. The web tool can be freely accessed at https://temporal-severe-covid.streamlit.app/, accessed on 16 November 2023.

Table 1 summarises the gene sets used by others to characterize MDSCs. In addition, based on our study where daily transcriptomic profiling was performed on six severe COVID-19 patients [43], we annotated the transcripts (in bold) that were temporally regulated during severe COVID-19 progression and recovery (Table 1). As expected, the majority of genes characteristic of M-MDSCs and PMN-MDSCs were also differentially regulated during severe COVID-19 pathogenesis (Table 1). To allow users to query the temporal changes in genes involved in severe COVID-19 pathogenesis, we also created a web tool (https://temporal-severe-covid.streamlit.app/, accessed on 16 November 2023) where users can input a list of genes or a list of MDSC-related gene sets from Table 1 to plot the normalized expression values across different time points before and after the respiratory nadir (Figure 2). Users can also run the web tool locally using the codes provided on GitHub (https://github.com/kuanrongchan/temporal_severe_covid, accessed on 16 November 2023). We believe that utilizing these gene sets rather than individual genes will enable users to better characterize the different MDSC subsets, which will be useful for single-cell RNAseq or bulk RNAseq data analysis. Future studies that systematically track these gene expression changes during the development of MDSCs will be instrumental in characterizing MDSC subsets in a more precise manner.

**Table 1:** Gene sets or gene signatures identified or used for identifying M- or PMN-MDSCs in the context of COVID-19. Genes in bold were temporally regulated during COVID-19 progression. Users can query the temporal dynamics of specific genes or MDSC-related gene sets during severe COVID-19 progression and resolution using the web tool at https://temporal-severe-covid.streamlit.app/, accessed on 16 November 2023.

M- or PMN-MDSC	Gene Sets Used/Signature Genes Identified	Reference
M-MDSC	**S100A8**, **S100A12**, **LGALS1**, **VCAN**, RETN, LYZ, **PLAC8**, MNDA, **CTSD**, **SELL**, STXBP2, **CYP1B1**, VIM, **CLU**, NKG7, **ALOX5AP**, NCF1, **MCEMP1**, **TIMP1**, SOD2, **CD163**, **NAMPT**, FAM65B, **ACSL1**, **VAMP5**, **LILRA5**, **VNN2**, ANXA6, IL1R2, **CALR**	[44]
	**CSTA**, **IL1B**, **HBB**, **LRG1**, **CXCR2**, **PROK2**, **IGFBP6**, HBA2, **SOCS3**, ASPRV1, C19orf12, GRINA, CSF3R, **CCR1**, **IFITM2**, **HP**, **CTSD**, TSPO, **S100A11**, **JUNB**, CYP4F2, CD84, **CLEC4E**, MAP1LC3B, GCNT2, GDA, **DUSP1**, **HDC**, **C5AR1**, **MSRB1**, **SRGN**, SELPLG, BTG1, MXD1, HIST1H2BI, **STEAP4**, **SLC40A1**, ARG2, SLPI, **CLEC4D**, **TXN**, FABP5, CDKN2D, **UPP1**, PRTN3, CHI3L1, TPD52, CXCL3, RNF149, GPCPD1, S100A6, **GSR**, **LMNB1**, **TACSTD2**, MTUS1, LITAF, **NPL**, **C19orf38**, **SEPHS2**, IL36G, **GLIPR2**, EIF4EBP1, F10, **VCAN**, PI16, SMPDL3A, GAPDH, ATP6V1G1, **FCGR2A**, PLA2G7, CD300LF, **YPEL3**, KIAA1551, **ADIPOR1**, **UBA52**, GLRX, **SELL**, **ATG3**, **ABTB1**, FGL2, **ALOX5AP**, **GADD45A**, **CD14**, **SIGLEC9**, **FBXL5**, **MYD88**, TGFBI, SNAP23, **PILRA**, FXYD5, **BST1**, **FLOT1**, **LILRB4**, PPT1, MRGPRX3, **R3HDM4**, **PKM**, PICALM, **SORL1**, **ZYX**, **ATP11B**, **RGS3**, CTSC, PTPN1, **TALDO1**, **STK17B**, **ANKRD33B**, **GYG1**, **IL4R**, **IER3**, NUDT4, SKAP2, IL13RA1, LILRB3, GATM, **NCF4**, **F13A1**, CDK2AP2, EMB, **GCLM**, **RAB24**, CD300LD, SERP1, CEBPB, **ALDOA**, **UBE2H**, TARM1, TSC22D3, CARHSP1, HEBP1, KLF13, **RBM3**, ELP1, **PGD**, ENO1, DHRS7, **OGFRL1**, **GDPD3**, SFXN5, **PYGL**, ANXA11, **DCK**, **OSM**, SDCBP, **UBB**, **EDEM2**, **LDHA**, UBALD2, **CCRL2**, FTH1, PKIB, **PNPLA2**, CAP1, KLF2, RND1, LCP1, ANXA2, HOPX, CAMK2D, SUPT4H1, GNG12, ARID5A, CD302, CSF2RB, PPP1R2, RAC2, SLC28A2, **PIM1**, **MPP1**, **RNASEL**, TONSL, **SSU72**, **TBC1D14**, ID2, **SPI1**, **HIST1H1C**, **IER2**, MMP8, **RTP4**, TMEM14C, UBE2B, **GPR146**, **PGLYRP1**, MBOAT7, **LAMP2**, BIN3, **PLSCR1**, EGR1, **EMILIN2**, **C5orf30**, TREM1, BIN2, CALM3, CBR3, SLFN12L	[45]
	**IFITM1**, **IFITM2**, **IFITM3**, GRINA, **HLA-DRA**, HLA-DQB1, **HLA-DPA1**, CD74	[46]
PMN-MDSC	**SAMD9L**, **IL1RN**, CSF3R, CSF1, GDA, **HP**, IFIT1, **IFIT3**, NUDT4, **IFIT2**, **NDST1**, **IL18RAP**, **PGLYRP1**, **CD177**, **ZBP1**, RSAD2, IL1R2, **MCEMP1**, TYROBP, SLPI, **CRISPLD2**, S100A6, CMPK2, **PADI4**, **RAF1**, **S100A9**, **S100A8**, SLC27A4, **PFKFB4**, **C5AR1**, **FPR1**, **FPR2**, **ADIPOR1**, **RASGRP4**, HCAR2, CD300LF, IP6K1, **PAG1**, **CCR1**, **NBEAL2**, **ISG15**, **BST1**, **ABTB1**, **SELL**, TREML2, TARM1, **DMXL2**, **LCN2**, **FBXL5**, MXD1, **RTP4**, TINAGL1, **SLC2A3**, **PYGL**, **LYST**, SLC2A6, **TIMP2**, **FFAR2**, **UPP1**, CD33, CERS6, SH2D3C, MMP8, **MMP9**, **DUSP6**, **RHOB**, **IL17RA**, FGR, MMP13, **OAS2**, **IL1B**, PECAM1, **IRF7**, CD44, **TLR2**, **LILRA6**, **SLFN5**, **PTAFR**, CXCR4, SAMHD1, THBS1, **STX11**, KCTD20, **CXCR2**, CCL3, EGLN3, **GABBR1**, SEMA4D, **GSR**, **OSM**, ASPRV1, KLF2, **LRG1**, PI16, SMAP2, AGRN, **YPEL3**, **MSRB1**, GRINA	[47]
	**S100A12**, ARG1, **CD177**, **MCEMP1**, and **GYG1**	[48]

## 4. Implications of MDSCs in COVID-19 Pathogenesis

SARS-CoV-2, as with other viral infections, perturbs normal hematopoiesis, especially in severe COVID-19 patients [49]. Besides demonstrating that the early upregulation of *MCEMP1* and downregulation of *HLA-DRA* expression occurred at least 4 days before the nadir of respiratory function in severe COVID-19 patients [17], emerging studies have also shown an increased accumulation of cells sharing features of M- [46] and PMN-MDSCs [48,50,51,52,53] in severe COVID-19 patients, suggesting the importance of these dysregulated myeloid cells in SARS-CoV-2 pathogenesis and disease progression [52]. The levels of M-MDSCs were detected more abundantly in the blood of severe COVID-19 patients rather than in the nasopharyngeal and endotracheal aspirates [54], which is consistent with findings showing that the gene signatures of MDSCs were detected more strongly in CD14-positive cells found in peripheral blood compared to immune cells in the bronchoalveolar lavage fluid [17]. Since the MDSC gene signature is prominent in the peripheral blood of severe COVID-19 patients, several other groups have also explored the prognostic potential of MDSCs. For instance, in a study conducted on 41 COVID-19 patients who have yet to progress to peak disease severity, circulating levels of M-MDSCs obtained within two weeks of symptom onset were found to predict disease severity [54]. In another study, CPT1a^+^VDAC1^+^HLA-DR^−^ M-MDSC frequencies were found to be significantly higher in severe compared to mild COVID-19 patients [55], and these differences were observed before the respiratory nadir in severe COVID-19 patients [17]. Other studies have also reported that M-MDSC levels were correlated with nasopharyngeal SARS-CoV-2 viral load [56] and associated with secondary infections and mortality in severe COVID-19 patients [57]. Interestingly, survivors of severe COVID-19 have lower PMN-MDSCs in their blood in comparison to the deceased group [58], which is in agreement with other evidence showing the correlation of higher PMN-MDSC frequency with increased mortality or disease fatality [19].

Besides showing an association of MDSC frequencies with disease severity, accumulating evidence now suggests that COVID-19-patient-derived MDSCs can exhibit immunosuppressive activities on T cells [19,59]. Specifically, M-MDSCs derived from COVID-19 patients have been shown to suppress the proliferation and inhibit the release of IFN-γ by T cells in an Arg-1-dependent manner [54]. In addition, ROS levels and the capacity of neutrophils to generate ROS are also significantly associated with disease severity [60,61], indicating the likely contribution of ROS in T cell suppression during severe COVID-19. In another study that performed a co-culture of patient-derived MDSCs with autologous MDSC-depleted PBMCs, it was found that MDSCs can promote the expansion of T_reg_ precursors while suppressing mature T_reg_ proliferation [62]. Thus, T cell suppression could also be mediated by regulating T_reg_ frequencies, which can consequently influence the outcome of SARS-CoV-2 infection [63]. Given that the early induction of functional SARS-CoV-2-specific T cell responses correlates with rapid viral clearance and mild disease in COVID-19 patients [64], these studies collectively indicate that the early expansion of MDSCs serves as a pro-viral strategy to evade early T cell detection. By expanding MDSC populations during the early stages of infection, viruses may hamper T cell activation and effector functions, thereby impairing antiviral responses. This evasion strategy potentially enables the virus to establish a foothold and replicate sufficiently before the immune response is induced for viral clearance. However, the precise role of MDSCs in viral infection remains an area of active research; the specific mechanism and implication of their interaction with immune cells remain poorly defined. Further studies are thus warranted to elucidate the complex interplay between MDSCs, T cells, and SARS-CoV-2.

## 5. Role of Inflammatory Responses to MDSC Development in Severe COVID-19

Besides the contribution of arginine depletion, ROS generation, and T_reg_ cells in MDSC development and T cell suppression, an increasing number of studies have also highlighted the role of inflammatory responses in shaping MDSC development. Viral proteins, such as the S protein, have been shown to induce the release of proinflammatory cytokines in human macrophages and lung epithelial cells via the TLR2-NF-κB pathway [65]. Other viral components, such as E protein [66], N [67], and ORF7a [68], have also been shown to induce inflammatory cytokine production through the activation of the inflammasome and NF-κB pathway [69,70]. The incubation of hematopoietic stem and progenitor cells (HSPCs) with plasma from severe COVID-19 patients induced MDSC-like cells [44], indicating that the pro-inflammatory responses triggered by infection can directly influence myeloid cell development. Among the different pro-inflammatory responses triggered by SARS-CoV-2 infection, IL-6 has been shown to be consistently elevated in the blood of severe COVID-19 patients [11,51,71] and can induce HSPCs [44] and human primary monocytes to develop into MDSC-like cells [72]. In other cases, blood levels of GM-CSF [73] and M-CSF [74] were significantly heightened in severe COVID-19 patients and a combinatorial treatment of HSPCs with IL-6, GM-CSF, and M-CSF enhanced the generation of MDSC-like cells [72]. Other cytokines, such as IL-8 [50,75], TNF-α, and IL-1β, were also observed to be markedly increased in the blood of severe COVID-19 patients [11] and were found to be significantly correlated with MDSC levels, possibly recruiting these cells to peripheral blood.

**Figure 3:**
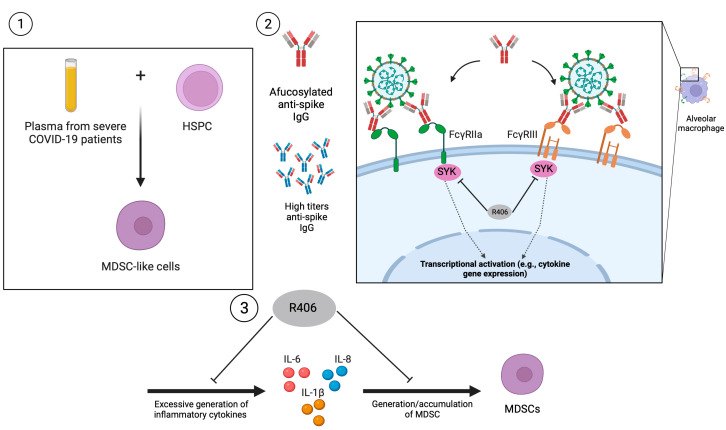
Potential mechanism for elevated MDSC levels in COVID-19 patients as suggested by three separate studies. (**1**) Reyes et al. (2021) [44] showed that the incubation of human HSPCs with plasma from severe COVID-19 patients, which contains high levels of the proinflammatory cytokine, IL-6, induced the generation of MDSC-like cells. (**2**) Hoepel et al. (2021) [77] demonstrated that high titers of anti-spike IgG or afucosylated anti-spike IgG from severe COVID-19 patients could bind to FcγRIII and FcγRIIa which activates Syk and led to enhanced transcription of proinflammatory cytokines such as IL-6. These effects were successfully abrogated by R406, the active component of fostamatinib, a Syk inhibitor. (**3**) Wigerblad et al. (2023) [78] performed a clinical study showing that R406 treatment decreased proinflammatory cytokine production and reduced generation of MDSCs, alongside other beneficial effects such as ameliorated interferon response in severe COVID-19 patients. MDSC: Myeloid-derived suppressor cells. HSPC: hematopoietic stem and progenitor cells.

In addition to viral proteins, high levels of anti-spike IgG or afucosylated anti-spike IgG after SARS-CoV-2 infection can trigger a heightened proinflammatory response [76] in human alveolar macrophages, which promotes the release of cytokines such as IL-6 and TNF via the IFN-signaling pathways [77]. The addition of the Syk inhibitor, R406, can ameliorate the release of proinflammatory cytokines, highlighting the role of Syk-mediated signaling in inducing pro-inflammatory responses following SARS-CoV-2 infection. Furthermore, the ability of R406 to impede the production of proinflammatory cytokines raises the question of whether R406 may also impact MDSC development, as pro-inflammatory responses are known to promote MDSC development. Indeed, a separate study demonstrated that R406 could restore myeloid homeostasis by reducing PMN-MDSC levels, increasing levels of monocytes with the HLA-DR^hi^ phenotype, and inhibiting the expression of antiviral genes [78]. However, more studies will need to be performed to establish the link between SARS-CoV-2 and Syk-signaling, and their interaction with MDSC development and severe disease outcome. A summary of the mechanisms involved is detailed in Figure 3.

## 6. Long-Term Changes in MDSCs after Severe COVID-19 Resolution

Given the short lifespan of MDSCs [21], as well as the clearance of SARS-CoV-2 and inflammation after disease resolution, most individuals are expected to experience a decline and normalization of MDSC levels [79]. However, intriguingly, there have been studies reporting persistent or elevated levels of MDSCs and myeloid progenitor cells [80,81,82,83,84], even in asymptomatic convalescents [85]. These observations suggest that there may be epigenetic reprogramming in the myeloid cells that allows MDSC persistence in recovered severe COVID-19 patients. Indeed, in a study by Cheong and colleagues [80], ATAC-seq and single-nuclei RNAseq collectively uncovered that monocytes and HSPCs from severe COVID-19-recovered patients exhibited phenotype and transcriptional programs characteristic of inflammation and myeloid activation. Notably, the increased myelopoiesis and epigenetic alterations could be observed for as long as 12 months following acute infection [80]. While the precise mechanism remains to be fully elucidated, these effects appear to be modulated by IL-6 as the IL-6 blockade led to abrogation of these observations [80]. Thus, in addition to suppressing hyper-inflammatory responses, IL-6 inhibitors such as tocilizumab may be useful in restoring myeloid dysregulation. Consistent with this hypothesis, a clinical study of over 300 participants revealed that tocilizumab treatment restored myeloid dysregulation and ameliorated inflammation compared to the placebo patient group [47]. However, it is important to note that, in another study involving 10 severe COVID-19 patients, tocilizumab did not affect MDSC levels [86], suggesting that other factors may be crucial in governing MDSC levels. These may include other key regulators such as HIF1α [87] and PGE2 [88,89], which are known to be upregulated in severe COVID-19 patients.

As MDSC levels can persist following recovery from severe COVID-19, it is possible that MDSCs may also contribute to long-COVID [90], where patients experience myriad lingering symptoms following recovery. Indeed, increased levels of immunoregulatory immune cells, such as T_reg_ cells [91] and MDSCs [82,83,85,92], have been observed to last up to one year following recovery in some long-COVID or COVID-19-recovered patients [81]. Notably, some COVID-19 convalescents possess elevated blood levels of IL-6 [82], although the significance of these findings in MDSC development and long-COVID has not been completely resolved [86].

## 7. Potential Therapeutic Drugs for Ameliorating MDSC Levels in COVID-19 Patients

Given the implication of MDSCs in severe COVID-19, targeting MDSCs may be a rational option for COVID-19 and possibly other infectious diseases that rely on MDSCs to facilitate pathogenesis. Therapeutics that induce MDSC apoptosis or eliminate the stimuli that promote MDSC generation [93] are some of the proposed ways to limit the effects of MDSCs on severe COVID-19 progression and disease pathogenesis. Some of these drugs have been shown to abolish the excessive production of proinflammatory cytokines and reduce MDSC levels in ex vivo and in vitro studies. For instance, the direct treatment of monocytic/T cell co-culture with 5-fluorouracil led to lower levels of cytokines such as IL-8 in the culture supernatant [86]. The removal or inhibition of stimuli known to favor MDSC generation may also be a potential therapeutic option. As S100A8/A9 is a stimulus favoring MDSC generation that is increased following SARS-CoV-2 infection, an inhibitor of S100A8/A9, paquinimod, has been proposed to reduce viral load and levels of aberrant immature neutrophils in mouse models [94]. Finally, as MDSCs can exert their immunosuppressive effects through the deprivation of amino acids important for T cell survival and function, the functional effects of MDSCs may potentially be perturbed by immunometabolic regulation such as amino acid supplementation. For example, the supplementation of amino acids such as L-arginine has been shown to be clinically beneficial for severe COVID-19 patients by reducing the length of hospitalization and requirement for respiratory support [95]. However, while these findings are promising, clinical trials will be required to ascertain the efficacy and side effects of these therapeutic strategies in reducing the burden of severe COVID-19 and long COVID, which may offer valuable insights into the contribution of MDSCs to severe COVID-19 pathogenesis.

## 8. Conclusions

Mounting evidence from multiple research groups hints at the mechanism of early MDSC expansion in severe COVID-19 patients and supports the role of MDSCs in suppressing antiviral and T cell immune responses against SARS-CoV-2. However, several questions remain unanswered, for instance, regarding the underlying mechanisms involved in the persistence of MDSCs in severe COVID-19 patients after recovery. As emphasized by Hegde et al. [24], it is also important that future efforts should emphasize the better characterization of MDSC states in order to allow for the easier detection of MDSCs for the early prognosis of severe COVID-19 and to enable a more in-depth understanding of the role that MDSCs play in severe COVID-19 pathogenesis.

## Data Availability

Not applicable.

## Abbreviations

MDSC	Myeloid-derived suppressor cells
ACE2	Angiotensin-converting enzyme 2
Arg-1	Arginase-1
ASC	Alanine-serine-cysteine
CMP	Common myeloid progenitor
CSF	Colony-stimulating factor
DAMP	Damage-associated molecular pattern
GM-CSF	Granulocyte-macrophage colony-stimulating factor
H_2_O_2_	Hydrogen peroxide
HSC	Hematopoietic stem cells
LOX1	Lectin-type oxidized LDL receptor 1
M-CSF	Macrophage colony-stimulating factor
M-MDSC	Monocytic MDSC
PD-1	Programmed cell death protein 1
PD-L1	Programmed death ligand 1
PMN-MDSC	Polymorphonuclear mdscs
ROS	Reactive oxygen species
SLC7A11	Solute carrier family 7 member 11
Syk	Spleen tyrosine kinase
Treg	Regulatory T cells
